# FDA-Approved Drugs that Protect Mammalian Neurons from Glucose Toxicity Slow Aging Dependent on Cbp and Protect Against Proteotoxicity

**DOI:** 10.1371/journal.pone.0027762

**Published:** 2011-11-16

**Authors:** Alex Lublin, Fumiko Isoda, Harshil Patel, Kelvin Yen, Linda Nguyen, Daher Hajje, Marc Schwartz, Charles Mobbs

**Affiliations:** 1 Fishberg Center for Neurobiology, Mount Sinai School of Medicine, New York, New York, United States of America; 2 Program in Gene Function and Expression, University of Massachusetts Medical School, Worcester, Massachusetts, United States of America; Catholic University Medical School, Italy

## Abstract

Screening a library of drugs with known safety profiles in humans yielded 30 drugs that reliably protected mammalian neurons against glucose toxicity. Subsequent screening demonstrated that 6 of these 30 drugs increase lifespan in *C. elegans*: caffeine, ciclopirox olamine, tannic acid, acetaminophen, bacitracin, and baicalein. Every drug significantly reduced the age-dependent acceleration of mortality rate. These protective effects were blocked by RNAi inhibition of *cbp-1* in adults only, which also blocks protective effects of dietary restriction. Only 2 drugs, caffeine and tannic acid, exhibited a similar dependency on DAF-16. Caffeine, tannic acid, and bacitracin also reduced pathology in a transgenic model of proteotoxicity associated with Alzheimer's disease. These results further support a key role for glucose toxicity in driving age-related pathologies and for CBP-1 in protection against age-related pathologies. These results also provide novel lead compounds with known safety profiles in human for treatment of age-related diseases, including Alzheimer's disease and diabetic complications.

## Introduction

A promising implication of aging research is that interventions directed at fundamental processes of aging may mimic the broad protective effects of dietary restriction against age-related pathologies (e.g., activation of sirtuins by resveratrol [1]). Furthermore, the discovery that specific drugs increase lifespan has led to new insight of the role the targets of these drugs play in the aging process. For example, screening drugs from a variety of functional classes and structure led to the discovery that anti-convulsant drugs extend lifespan in *C. elegans*, supporting an unexpected role of neuronal activity in determining lifespan [2]. Similarly, a screen of over 88,000 compounds led to the discovery that the anti-depressant drug mianserin extends lifespan in *C. elegans*, supporting a novel role for serotonin in determining lifespan [3]. Other studies in *C. elegans* on the role of the TOR pathway in determining lifespan [4] led to the discovery that rapamycin extends lifespan in mice [5], crucially supporting that the mTOR pathway plays a key role in mammalian aging and providing a lead compound for a variety of age-related diseases, especially cancer.

We have recently reported that the protective effects of dietary restriction and ablation of the insulin-like pathway require induction of the transcriptional co-activator Creb-binding protein (Cbp), whose expression in the hypothalamus of mice also accounts for over 80% of lifespan variance in 5 strains of mice [6]. This study also suggested that the protective effects of dietary restriction and Cbp are mediated by a metabolic shift away from glucose utilization and toward beta oxidation [6]. These and other observations [7], [8] suggest that drugs which protect against glucose toxicity would plausibly mimic many of the protective effects of dietary restriction, including a reduction in age-dependent acceleration of mortality rate [9]. We also hypothesized that the protective effects of such drugs would depend on CBP-1 and possibly DAF-16 [6].

To address this hypothesis we developed a high-throughput screen to discover drugs that protect against glucose toxicity in mammalian neurons, since, as indicated above, growing evidence indicates that neurons play a key role in aging and the protective effects of dietary restriction [10]. We focused on a library of drugs with established safety profiles in humans, mainly drugs approved for use in humans by the FDA, since such drugs would be most readily translated for clinical use [11]. Drugs corroborated to be protective in mammalian neurons were then screened for activity to increase lifespan, reduce age-related acceleration of mortality rate, and reduce pathology in a transgenic model for proteotoxicity in Alzheimer's disease (CL2006) [12]. Finally, we assessed if protective effects of these drugs depend on DAF-1*6* or CBP-1.

## Results

### Thirty drugs that protect against glucose toxicity in mammalian neurons

Many lines of evidence suggest that glucose metabolism and toxicity contribute to both aging [8] and diabetic complications [13]. We therefore hypothesized that drugs which ameliorate glucose-induced vulnerability to oxidative stress would also protect against toxicity during aging. To discover such drugs we developed an assay to assess glucose-induced toxicity in neurons [14]. The primary screen, utilizing dye-based assay for neuronal viability, yielded 42 drugs that significantly enhanced viability at 15 mM glucose (p<0.05, >50% enhanced viability). These 42 protective drugs were then screened using a secondary assay, measuring lactate dehydrogenase released into the medium as a marker of cell death. Thirty of these 42 drugs significantly reduced neuronal death at 15 mM glucose (Table 1). Dose-response curves were then generated for each drug, which corroborated that all drugs enhanced neuronal viability at 15 mM glucose in the presence of oxidative stress (Table 1 and Figure S1).

**Table 1:** Drugs that protect against glucose induced hydrogen peroxide toxicity in mouse primary neurocytes.

Drug	%LDH (Mean)	%LDH (Sem)	EC 50	Human dosage	LD 50
CAFFEINE	26.0	6.5	14.1	100–200 mg/4 hrs	192 mg/kg oral
CICLOPIROX OLAMINE	17.5	4.7	1.1	NA	NA
TANNIC ACID	27.1	6.2	0.17	NA	1000 mg/kg oral
ACETAMINOPHEN	47.4	8.7	8.2	90 mg/kg/day	1944 mg/kg oral
BACITRACIN	27.1	7.6	34.6	NA	>3750 mg/kg oral
BAICALEIN	47.8	16.1	18.6	NA	NA
HYDRALAZINE HCl	33.5	14.3	92.3	7.5 mg/kg max	173–187 mg/kg oral
POTASSIUM p-AMINOBENZOATE	15.4	6.5	5.2	NA	NA
AMILORIDE HCl	23.4	2.4	18.2	5–10 mg/day	36–85 mg/kg oral
METHACYCLINE HCl	23.8	5.2	53.8	8.5 mg/kg/day	252 mg/kg IP
2-THIOURACIL	27.3	11.2	23.6	30–60 mg/day	2100 mg/kg oral
AMINOGLUTETHIMIDE	31.0	13.2	32.3	250mg/6 hrs	625 mg/kg IP*
PROTHIONAMIDE	32.0	12.1	5.8	1000 mg/day	1320 mg/kg oral
AESCULIN	32.3	7.6	44.8	100–150 mg/day	NA
PINDOLOL	33.6	5.4	5.1	10–60 mg/day	263 mg/kg oral
TOLAZAMIDE	33.6	5.4	13.1	100–1000 mg/day	200 mg/kg oral*
BERBAMINE HCl	35.4	8.9	88.8	150 mg/6 hrs	15000 mg/kg oral
FENBUFEN	35.7	5.3	42.6	4.285 mg/kg/8 hrs	800 mg/kg oral
BETAMETHASONE	38.5	11.5	62.5	.6–7.2 mg/day	1607 mg/kg oral*
BACLOFEN	39.0	0.6	95.7	40–80 mg/day	145 mg/kg oral
3,5-DINITROCATECHOL	39.8	8.0	60.6	NA	500 mg/kg oral*
PIPERINE	42.3	8.3	69.5	10 mg/day	514 mg/kg oral
CHLORZOXAZONE	43.1	3.7	85.5	250–750 mg/6 hrs	763 mg/kg oral
BUSULFAN	43.2	12.6	10.3	2–4 mg/kg/day	15 mg/kg oral
BITHIONOL	43.7	10.6	88.0	5–25 mg/day	7 mg/kg oral
AMPROLIUM	44.6	5.6	14.4	NA	6170 mg/kg oral*
DIHYDROJASMONIC ACID	45.3	2.6	95.2	NA	NA
TRANYLCYPROMINE SULFATE	46.3	10.2	102.5	30–60 mg/day	30 mg/kg IP
ANTIPYRINE	49.3	21.5	86.8	NA	1750 mg/kg oral
SODIUM p-AMINOSALICYLATE	56.5	5.7	10.0	5–6 g/12 hrs	8000 mg/kg oral
ETODOLAC	77.7	2.7	88.3	1000 mg/day	94 mg/kg oral

The first seven drugs represent candidates that also have positive effects in *C. elegans* models. The remaining dugs are ranked according to their effectiveness in comparison to untreated controls (%LDH (mean)). The effective concentration in culture is represented as “EC 50.” When available, normal human dosages and lethal mammalian dose (LD 50) in rat, unless stated otherwise, are listed. Chemical formula, molecular weight, and current uses for the drugs (Function) are shown.

*LD 50 performed in mouse.

### Caffeine increases lifespan and slows aging dependent on DAF-16 and CBP-1 and delays proteotoxicity

**Figure 1:**
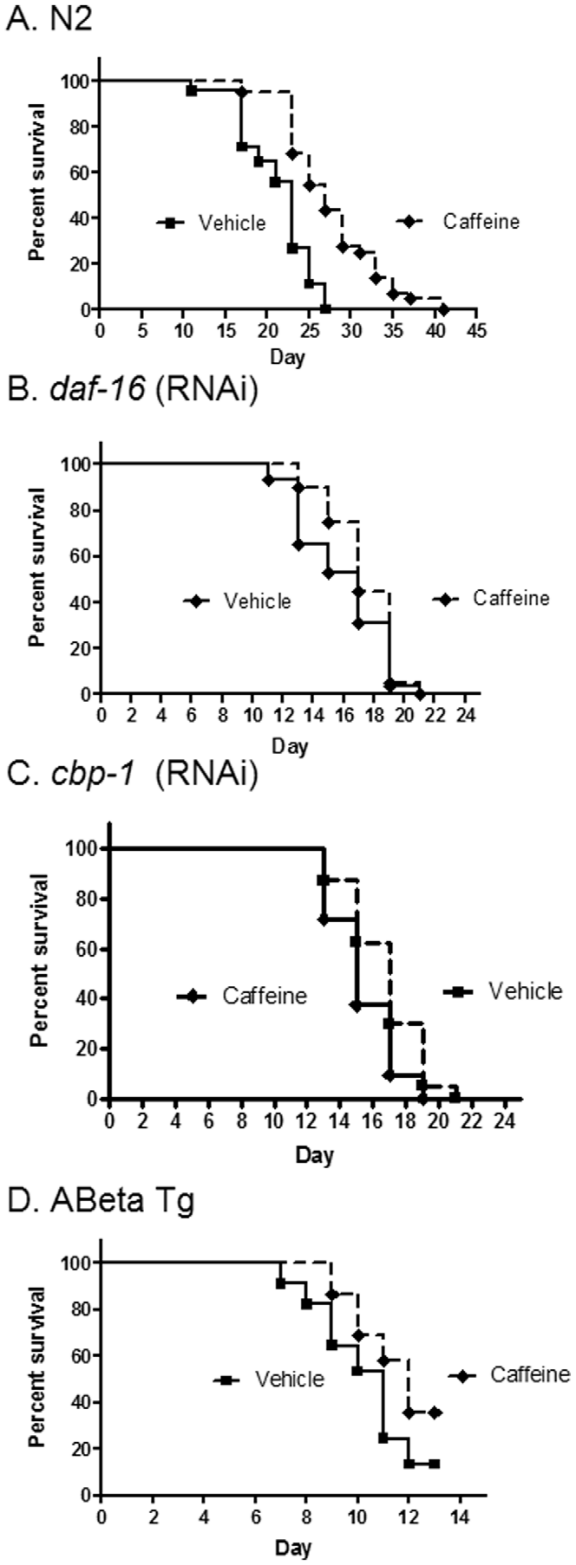
Caffeine increased maximum and median lifespan dependent on DAF-16 and CBP-1 and delays toxicity in a transgenic model of Alzheimer's disease. A. Caffeine (0.1%) increases maximum and median lifespan (P<0.01). B. Caffeine does not increase lifespan when *daf-16* is inhibited by RNAi. C. Caffeine does not increase lifespan when *cbp-1* is inhibited by RNAi. D. Paralysis is delayed and survival of the Abeta Tg is increased with 0.1% caffeine vs. vehicle (P<01).

To assess if these drugs would be protective during aging, effects of each drug on lifespan were assessed in adult N2 *C. elegans*, at concentrations of 0.01, 0.1, and 1%. Seven of the thirty drugs produced significant increases in lifespan for at least one concentration (often the drugs increased lifespan at one concentration and reduced lifespan at a higher concentration). After the initial screen indicated that at least one concentration of these drugs increased lifespan, the protective effects of each drug at the optimum dose was corroborated using at least two more detailed analyses of survival curves in wild-type worms as well as in the Abeta transgenic model of Alzheimer's disease proteotoxicity. For example, caffeine at 0.1% increased maximum lifespan by 52% (Figure 1A) (Table 3) and also significantly increased median lifespan (p<0.01 by log rank Mantel-Cox test; n = 45) (Figure 1A). Protective effects of caffeine were completely blocked by inhibition of either DAF-16 (Figure 1B) or CBP-1 (Figure 1C) by RNAi. Finally, 0.1% caffeine also significantly delayed pathology in a transgenic Abeta *C. elegans* model of proteotoxicity in Alzheimer's disease (p<0.01; n = 30). Caffeine also reduced age-dependent acceleration of mortality rate by 53%, and this protective effect was also blocked by RNAi inhibition of CBP-1 or DAF-16 (Table 2).

**Table 2:** Average G-value gathered by maximum likely-hood estimates methods.

	a(x10^5^)	g(X10^2^)
N2 Vehicle (n = 45)	25.58	30.55
N2 Caffeine (n = 45)	102.73	16.73*
*daf-16*(RNAi) Vehicle (n = 32)	34.96	40.83
*daf-16*(RNAi) Caffeine (n = 20)	1.71	56.89
*cbp-1* (RNAi) Vehicle (n = 40)	7.63	49.36
*cbp-1* (RNAi) Caffeine (n = 32)	8.75	53.32
N2 Vehicle (n = 45)	25.58	30.55
N2 CPX(n = 45)	24.59	20.27*
*daf-16*(RNAi) Vehicle (n = 32)	34.96	40.83
*daf-16*(RNAi) CPX (n = 30)	0.06	65.37*
*cbp-1* (RNAi) Vehicle (n = 40)	7.64	49.36
*cbp-1* (RNAi) CPX (n = 30)	0.26	65.48
N2 Vehicle (n = 45)	25.58	30.55
N2 Tannic Acid (n = 45)	152.53	16.26*
*daf-16*(RNAi) Vehicle (n = 28)	28.42	51.02
*daf-16*(RNAi) Tannic Acid (n = 15)	80.2	37.41
*cbp-1* (RNAi) Vehicle (n = 26)	32.74	58.39
*cbp-1* (RNAi) Tannic Acid (n = 24)	14.11	71.84
N2 Vehicle (n = 45)	115.72	18.66
N2 Acetaminophen (n = 45)	43.07	19.45
*daf-16*(RNAi) Vehicle (n = 32)	34.96	40.83
*daf-16*(RNAi) Acetaminophen (n = 30)	270.76	19.38*
*cbp-1* (RNAi) Vehicle (n = 40)	7.64	49.36
*cbp-1* (RNAi) Acetaminophen (n = 45)	0.6	63.51
N2 Vehicle (n = 45)	115.72	18.66
N2 Bacitracin (n = 45)	104.29	16.09
*daf-16*(RNAi) Vehicle (n = 32)	34.96	40.83
*daf-16*(RNAi) Bacitracin (n = 35)	265.53	20.20*
*cbp-1* (RNAi) Vehicle (n = 40)	7.64	49.36
*cbp-1* (RNAi) Bacitracin (n = 46)	4.22	56.53
N2 Vehicle (n = 45)	25.58	30.55
N2 Bacailene (n = 45)	68.57	17.34*
*daf-16*(RNAi) Vehicle (n = 29)	1.3	62.68
*daf-16*(RNAi) Bacailene (n = 28)	44.9	35.08*
*cbp-1* (RNAi) Vehicle (n = 29)	2.98	58.53
*cbp-1* (RNAi) Bacailene (n = 30)	2.82	65.1

*Significant decrease in values (as measured by one-sided chi squared).

The relative rate of aging as measured by Gompertz's analysis. The change in the initial or age- independent rate of mortality relative to vehicle treated controls (a). The change in age-dependent mortality rate, or Gompertz's variable (g) in relation to vehicle treated controls.

**Table 3:** N2 Lifespan.

Drug	Mean	Sem +/−	Min	Max	Number (n)
Vehicle	21.31	0.58	11	27	45
Caffeine	27.58	0.51	17	41	45
CPX	29.98	0.43	11	39	45
Tannic acid	26.60	0.50	17	43	45
Acetaminophen	31.67	0.43	23	47	45
Bacitracin	33.78	0.37	17	47	45
Baicalein	29.13	0.44	17	41	45

Lifespan of *C. elegans* (N2) in the presence of drugs administered at the optimal concentration. The average mean lifespan (in days) and the standard deviation were calculated from experiments (45 total worms per condition). Min and Max mark the first and last day (respectively) that an animal was scored as dead.

### Ciclopirox increases lifespan and slows aging, but does not delay proteotoxicity

**Figure 2:**
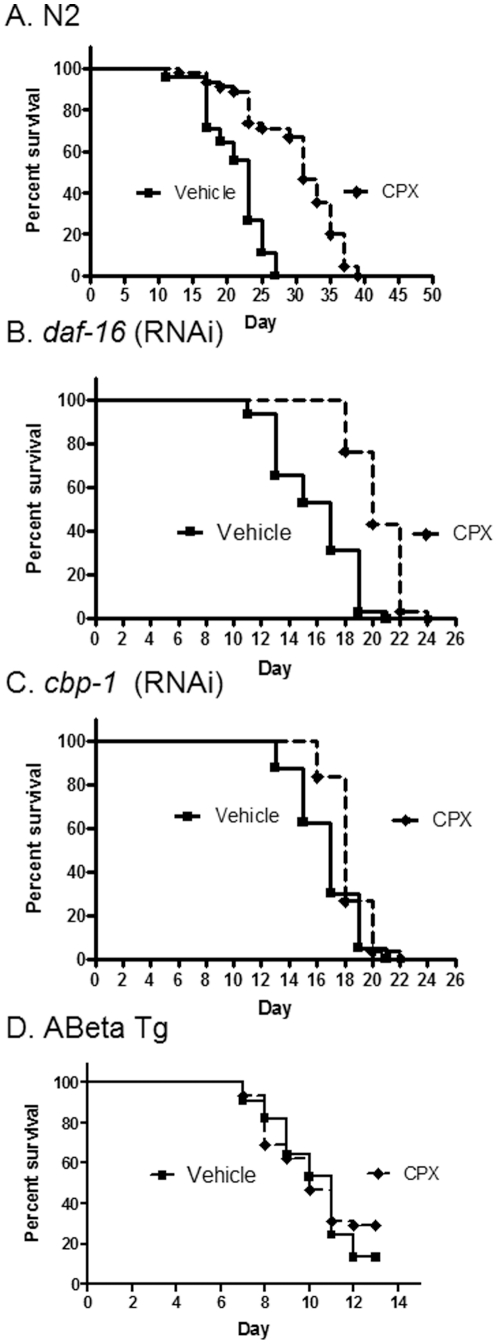
Ciclopirox olamine increases N2 longevity in a CBP-1 and DAF-16 independent, manner but fails to rescue A-beta toxicity. A. N2 longevity is increased with .01% ciclopirox olamine vs. vehicle (P<0.01). B. Survival of *daf-16* (RNAi) is increased with .01% ciclopirox olamine vs. vehicle (P<0.01). C. Survival of *cbp-1* (RNAi) is increased with .01% ciclopirox olamine vs. untreated control (P<.01). D. Survival of Abeta Tg is unchanged with .01% ciclopirox olamine vs. vehicle.

Ciclopirox olamine (0.01%), used to treat dermatological fungus infections, also increased maximum lifespan by 52% (Table 3) and significantly increased median lifespan (p<0.01 by Mantel-Cox log rank test; n = 45) (Figure 2A). In contrast to caffeine, ciclopirox increased lifespan even when *daf-16* was inhibited by RNAi (Figure 2B) (p<0.01; n = 30). Like the other drugs tested, *cbp-1* inhibition by RNAi blocked the effect of ciclopirox to increase maximum lifespan. However, unlike the other drugs in this study, the effect on median lifespan produced by ciclopirox, while reduced, was still significant (p<0.01; n = 30). Furthermore, like caffeine, ciclopirox reduced age-dependent acceleration of mortality rate (Table 2). Interestingly, inhibition of both *daf-16* and *cbp-1* not only blocked this protective effect but even appeared to unmask a toxic effect of ciclopirox to enhance age-related acceleration of mortality rate accompanied by a striking reduction in initial mortality rate (Table 2). A similar effect was observed for caffeine, though this did not achieve statistical significance (Table 2). In further contrast with caffeine, ciclopirox did not protect against pathology associated with proteotoxicity (Figure 2D), and in fact at the 1% concentration accelerated that pathology (not shown).

### Tannic acid increases lifespan and slows aging dependent on DAF-16 and CBP-1 and delays proteotoxicity

**Figure 3:**
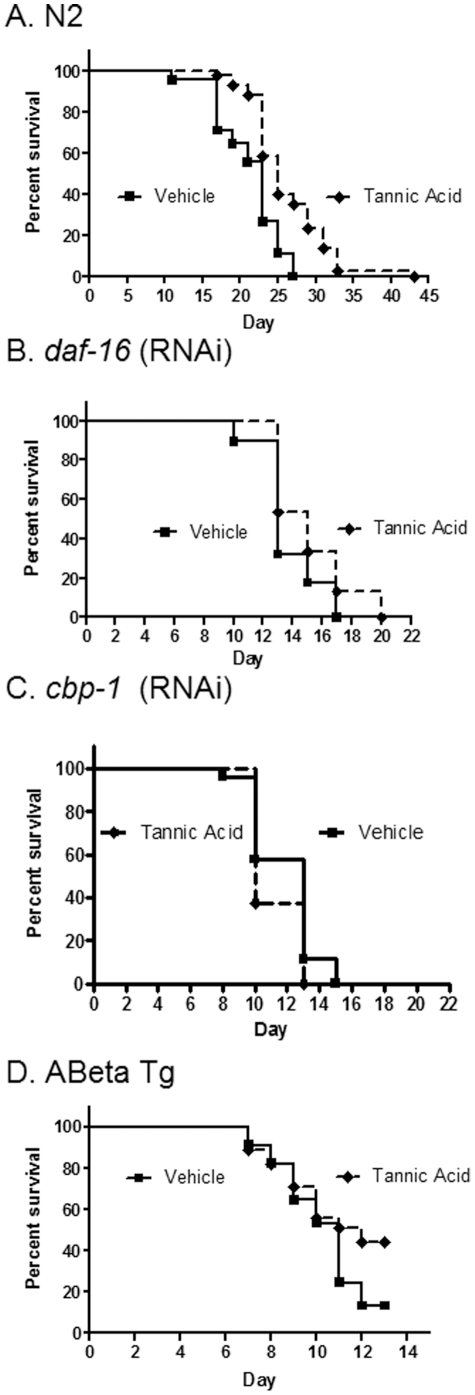
Tannic acid increases N2 longevity in a CBP-1 and DAF-16 dependent, manner and reduces Abeta toxicity. A. N2 longevity is increased with .01% tannic acid vs. vehicle (P<0.01). B. Survival of *daf-16* (RNAi) is unchanged with .01% tannic acid vs. vehicle. C. Survival of *cbp-1* (RNAi) is unchanged with .01% tannic acid vs. untreated control. D. Survival of A-beta Tg is increased with .01% tannic acid vs. vehicle (P = 0.01).

Tannic acid (0.01%), a highly soluble polyphenol, increased maximum lifespan by 59% (Table 3) and significantly increased median lifespan (P<0.01 by Mantel-Cox log rank test; n = 45) (Figure 3A). As with caffeine, inhibition of DAF-16 (Figure 3B) or CBP-1 (Figure 3C) blocked the effect of tannic acid to increase lifespan. As with caffeine and ciplopirox, tannic acid also reduced age-related acceleration of mortality rate, and inhibition of either and CBP-1 or DAF-16 prevented this protective effect (Table 2). Tannic acid also produced a striking delay in the onset of pathology associated with proteotoxicity, such that at day 13, only 15% of control worms remained mobile compared to 45% of worms treated with tannic acid (P<0.01; n = 30) (Figure 3D). However, tannic acid at 1% concentration significantly reduced lifespan.

### Acetaminophen increases lifespan dependent on CBP-1 and does not delay proteotoxicty

**Figure 4:**
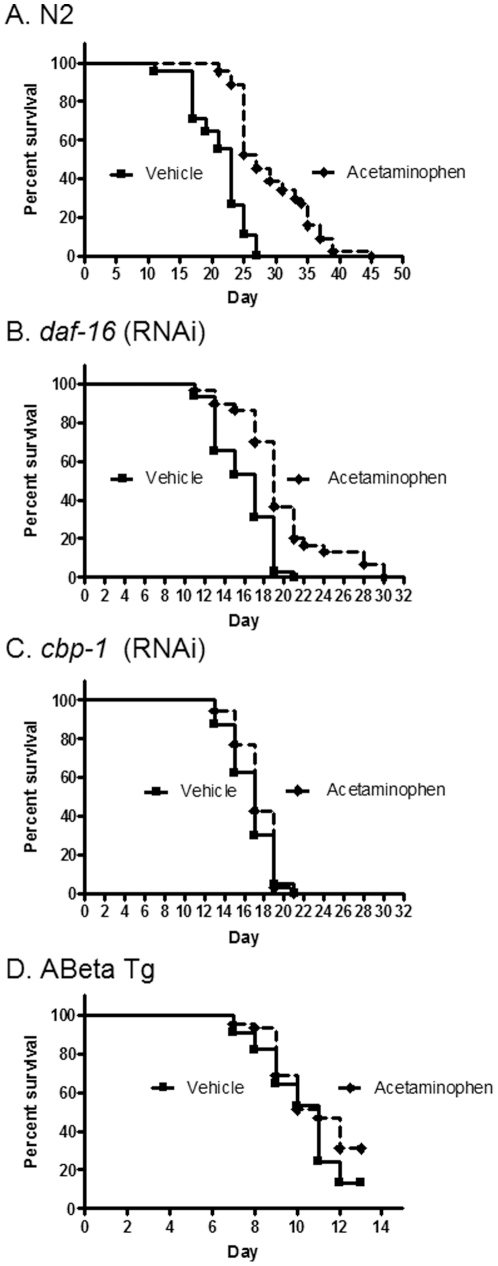
Acetaminophen increases N2 longevity in a CBP-1 dependent, but DAF-16 independent, manner but fails to rescue Abeta toxicity. A. N2 longevity is increased with 0.1% acetaminophen vs. vehicle (P<0.01). B. Survival of *daf-16* (RNAi) is increased with 0.1% acetaminophen vs. vehicle (P<0.01). C. Survival of *cbp-1* (RNAi) is unchanged with 0.1% acetaminophen vs. untreated control. D. Survival of Abeta Tg is unchanged with 0.1% acetaminophen vs. vehicle.

Acetaminophen (0.01%), a non-steroidal anti-inflammatory drug, increased maximum lifespan by 66% (Table 3) and significantly increased median lifespan (P<0.01; N = 45) (Figure 4A). Inhibition of DAF-16 did not prevent the effect of acetaminophen to increase lifespan (Figure 4B), but inhibition of CBP-1 completely prevented the protective effects of acetaminophen (Figure 4C). Acetaminophen differed from the drugs described above in that the extension of lifespan was not associated with reduction in acceleration of mortality rate under standard condition (Table 2). However, when *daf-16* was inhibited acetaminophen significantly reduced acceleration of mortality rate (Table 2), further supporting that the protective effect of this drug is independent of DAF-16. In contrast, when *cbp-1* was inhibited, acetaminophen if anything increased mortality rate, again supporting that the protective effects of acetaminophen depends on CBP-1. However, acetaminophen failed to delay the onset of pathology associated with proteotoxicity. At a higher concentration (1%) acetaminophen significantly reduced lifespan (not shown).

### Bacitracin increases lifespan dependent of CBP-1 and delays proteotoxcity

**Figure 5:**
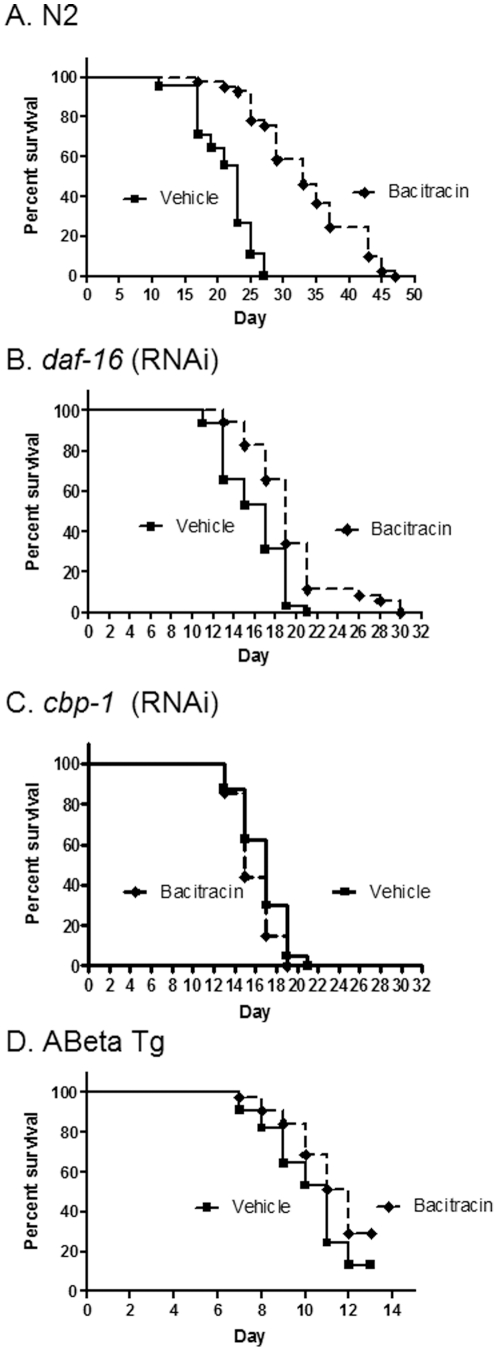
Bacitracin increases N2 longevity in a CBP-1 dependent but DAF-16 independent, manner and reduces Abeta toxicity. A. N2 longevity is increased with 01% bacitracin vs. vehicle (P<0.01). B. Survival of *daf-16* (RNAi) is increased with 01% bacitracin vs. vehicle (P = 0.01). C. Survival of *cbp-1* (RNAi) is unchanged with 01% bacitracin vs. untreated control. D. Survival of Abeta Tg is increased with 01% bacitracin vs. vehicle (P<0.02).

Bacitracin (1%), a topical antibiotic, increased maximum lifespan by 74% (Table 3) and significantly increased median lifespan (Figure 5A) (P<0.01; n = 45). Inhibition of DAF-16 reduced the life-extending effect of bacitracin but did not completely block it (Figure 5B; p<0.01; n = 35). In contrast, inhibition of *cbp-1* completely blocked the life-extending effect of bacitracin (Figure 5C). Like acetaminophen, bacitracin increased lifespan without significantly reducing age-associated acceleration of mortality rate (Table 2). However, bacitracin did reduce acceleration of mortality rate when *daf-16* is inhibited (which increased mortality rate), an effect not observed with *cbp-1* was inhibited (Table 2). These results further demonstrate that the protective effects of bacitracin are independent of DAF-16 but dependent on CBP-1. Bacitracin also significantly delayed pathology associated with proteotoxicity (P>0.02; n = 30).

### Baicalein increases lifespan and slows aging dependent on CBP-1 but does not significantly delay proteotoxicity

**Figure 6:**
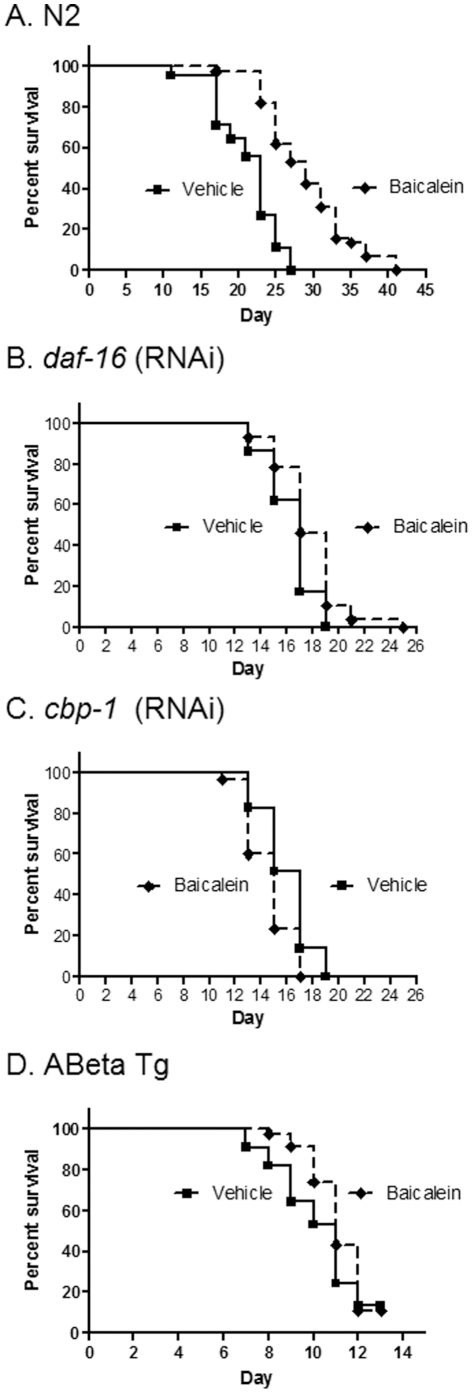
Baicalein increases N2 longevity in a CBP-1 dependent but DAF-16 independent, manner but fails to rescue Abeta toxicity. A. N2 longevity is increased with 0.1% baicalein vs. vehicle (P<0.01). B. Survival of *daf-16* (RNAi) is increased with 0.1% baicalein vs. vehicle (P = 0.01). C. Survival of *cbp-1* (RNAi) is reduced with 0.1% baicalein vs. untreated control (P<0.01). D. Survival of Abeta Tg is unchanged with 0.1% baicalein vs. vehicle.

Baicalein (0.1%), an anti-inflammatory component of a traditional Chinese herbal preparation [15], increased maximum lifespan by 52% and significantly increased median lifespan (Figure 6A; p<0.01; n = 45). Although inhibition of DAF-16 substantially reduced the protective effects of baicalein, the drug still significantly increased lifespan when *daf-16* was inhibited by RNAi (Figure 6B; p = 0.01; n = 28). In contrast *cbp-1* RNAi completely blocked the increase in lifespan produced by baicalein (Figure 6C). Interestingly, even though baicalein protects against Abeta toxicity in mammalian neurons [16], at the concentration that extended lifespan the compound did not significantly reduce pathology associated with Abeta proteotoxicity in *C. elegans* (Figure 6D).

## Discussion

In the present studies 1.5% of the drugs screened protected against glucose toxicity and a striking 23% of these drugs significantly increased lifespan. By comparison, in an unbiased screen of 88,000 compounds, only 0.13% of the drugs increased lifespan (half of which increased lifespan by less than 10%). Of the drugs that increased lifespan, 86% slowed the rate of aging, as indicated by analysis of mortality rate statistics [17] using a recently validated algorithm [18]. Inhibition of *cbp-1*blocked the protective effects of these drugs during aging just as it inhibits protective effects of dietary restriction (but not cold) [6]. In contrast only a few of the protective effects of these were dependent on DAF-16, just as the protective effects of only some methods of dietary restriction are dependent on DAF-16 [6], [19]. These observations are consistent with other evidence that glucose metabolism and toxicity play a prominent role in driving the aging process [7], [8], [20], as it does diabetic complications [13]. Similarly these studies support that a reduction in glucose toxicity mediates protective effects of dietary restriction during aging dependent on the activity of Cbp [6].

The present studies began with a blinded screen of the Spectrum Collection (Microsource) for potential protective effects against glucose toxicity in mammalian neuron and thus for potential use to prevent or reverse complications of diabetes. Similar screens of this library of compounds, most of which have known safety profiles for use in humans, have led to the potential repositioning of therapeutic drugs with known safety profiles for novel therapeutic purposes [11]. A major consideration when carrying out such screens is the challenge of obtaining the optimally protective dose, since protective compounds are generally toxic at higher doses. Indeed we observed in the initial screen in mammalian neurons that at the screening concentration many drugs were in fact toxic. Therefore we screened three concentrations in *C. elegans* and found that many of the drugs at the highest concentration examined (1%) reduced lifespan, whereas at lower concentrations some drugs increased lifespan. A related concern is the extent to which the protective concentrations observed in the *C. elegans* study, in which the compounds were added to the environment, would translate to doses that are safe to use in humans or other mammals. In the present study we screened at concentrations from 0.01 to 1% (roughly 100 uM to 10 mM) in the medium because such concentrations often produce similar effects in *C. elegans* as is produced at therapeutic doses in humans (e.g., fluoxetine [21]), although of course it is not clear precisely what is the tissue concentration of the drug in the worm under these conditions.

Since the safety profile of these protective drugs is known and in many cases mechanisms of protective action have been studied, they make promising lead compounds to treat age-related diseases including diabetes and Alzheimer's disease. Thus caffeine (ED50 = 12 mg/kg; LD50 = 200 mg/kg) was effective at a 0.1% concentration (5.1 mM) to increase lifespan and reduce proteotoxicity, corroborating a similar result that 3.6 mM caffeine added to the medium reduced pathology in the same transgenic model of proteotoxicity associated with Alzheimer's disease [22]. Many epidemiological studies have observed that consumption of coffee and other caffeinated drinks appears to be highly protective against a variety of pathologies including Parkinson's disease [23] and Alzheimer's disease [24]. Similarly, administration of caffeine in experimental models of these diseases is protective [25]. Many mechanisms have been suggested to mediate these protective effects of caffeine, but none have yet been established [24].

Ciclopirox is used clinically as a topical antifungal agent so an ED50 for oral doses is not known. However, the LD50 for oral doses is about 2000 mg/kg (e.g., 10-fold higher than for caffeine) and it was protective at a concentration 10-fold lower than for caffeine. Interestingly ciclopirox protects PC12 neuronal cells from cell death after removal of trophic support [26], and protects astrocytes from peroxynitrate toxicity by maintaining mitochondrial function, through unknown mechanisms. Tannic acid also exhibits an LD50 of about 2000 mg/kg and protects neurons [27] and produces many other protective effects; although many mechanisms have been suggested, none have been established [28]. Interestingly acetaminophen (LD50 about 1000 mg/kg) protects both neurons and brain endothelial cells against oxidative stress through a mechanism that may entail inhibition of apoptosis [29], [30]. Bacitracin (LD50 about 1000 mg/kg) is a topical antibiotic but also appears to specifically inhibit cholinesterases associated with plaques and tangles in Alzheimer's disease [31]. Although an antibiotic, the mechanism by which bacitracin increases lifespan is probably not mediated by dietary restriction secondary to reduced bacterial growth, since dietary restriction reduces the rate of aging in *C. elegans* [9] whereas bacitracin did not reduce the rate of aging (Table 2). Baicalein is an anti-inflammatory flavanoid isolated from the traditional Chinese herbal preparation known as huáng qín (derived from *Scutellaria baicalensis*) which has been shown to protect neurons against beta-amyloid toxicity [16] and other insults including ischemic stroke [32] through unknown mechanisms.

Thus all the compounds discovered in this screen have been shown to be neuroprotective under various circumstances but the only clear common denominator is that the mechanisms by which these diverse compounds exert their protective effects remain unclear. It is therefore of particular interest that the protective effects of each of these compounds is blocked by inhibition of *cbp-1* by RNAi whereas similar inhibition of *daf-16* only blocked protective effects of 2 of these compounds. Since RNAi does not completely block expression of DAF-16 in all cells this does not rule out that DAF-16 plays a role in mediating some of these protective effects. Nevertheless the evidence clearly supports a more robust role for CBP-1 than for DAF-16 in mediating protective effects of these drugs as it does in several protocols of dietary restriction [6]. Since the protective effects of these drugs was based on a screen for neuroprotection against glucose toxicity, against which CBP-1 also appears to be particularly protective [6], it is probably not a coincidence that the protective effects of these drugs are also largely dependent on CBP-1. On the other hand while 5 of the 6 drugs reduce the rate of aging and thus mimic the effects of dietary restriction (caffeine, ciclopirox, tannic acid, baicalein and, when *daf-16* is inhibited, bacitracin and acetaminophen). Similarly while 3 of the 6 drugs delayed pathology in a model of proteotoxicity associated with Alzheimer's disease (caffeine, bacitracin, and tannic acid), 3 did not. Thus, while the protective effects of all these drugs appear to depend on CBP-1, there are apparently other differences in mechanisms mediating the protective effects of these drugs.

In conclusion the present studies demonstrate that drugs which protect against glucose toxicity exhibit a remarkably high probability of increasing lifespan and reducing proteotoxicity. Furthermore these protective effects, like protective effects of dietary restriction and DAF-16, are highly dependent on the transcriptional factor CBP-1, but much less so, on DAF-16. Finally, the present studies greatly extend the number of corroborated drugs that may protect against a wide range of age-related pathologies including diabetic complications, Alzheimer's disease, and the rate of aging itself.

## Methods

### *C. elegans* strains

All strains were obtained from the Caenorhabditis Genetics Center, funded by the NIH National Center for Research Resources (NCRR), and maintained at 20° C, under standard conditions [33]. Strain CL2006, dvIs2[pCL12(*unc-54/human Abeta peptide 1-42 minigene) + pRF4*], used as a model for proteotoxicity in Alzheimer's disease, was created by Chris Link [12].

### Drug screen

The initial drug screen was from a compound library based on the Spectrum Collection (Microsource), as prepared and coded by the Juvenile Diabetes Research Foundation. The screen entailed assessing viability of primary (E16) cortical neurons after a 1-hour exposure to a low dose (30 uM) of hydrogen peroxide, using two distinct 96-well-based assays for neuronal viability. In contrast to some in vitro models of hyperglycemia, which show direct toxic effects of extremely high, non-physiological (often above 30 mM) levels of glucose, 15 mM glucose did not directly reduce neuronal viability, compared to 5 mM glucose. However, after a brief exposure to 100 uM hydrogen peroxide (determined by a dose-response curve), incubation at 15 mM glucose reduced neuronal viability 24 hours later by 75%, compared to 5 mM glucose. Twenty-four hours later, medium was removed and stored for subsequent assay of lactate dehydrogenase (Promega), or a viability assay (CKK-8; Dojindo Molecular Technologies). The wells were incubated and read by an ELISA reader at 450 nm, according to the manufacturer's instructions. Any drug producing a statistically significant increase in cell viability by the CCK-8 assay (n = 8 cells/drug at 15 mM glucose/100 uM hydrogen peroxide) was re-screened using the LDH assay as well as a subsequent replicate CCK-8 assay and dose-response curves. Following the screen in primary cortical neurons all drugs used in the *C. elegans* screens (caffeine, ciclopirox olamine, tannic acid, acetaminophen, baicalein, and bacitracin) were obtained from Sigma. Drug treatment for *C. elegans* entailed adding 400 ul of a solution containing either 0.01%, 0.1%, or 1% (by weight) of the designated drug, dissolved in 50% ethanol in water, to 10ml of solidified agar in a 6 cm petri dish. Controls agar plates were treated with 50% ethanol in water (vehicle). All drugs were added after overnight incubation at 37°C with bacterial strains: OP50, L4440 with RNAi cassette of the gene of interest, or L4440 (empty RNAi vector).

### Phenotypic assessment

Worms were scored every other day starting at day 3 of adulthood. Survival was assayed by prodding worms with a platinum wire and observing movement. Lack of movement was scored as death, following which the worm would be removed from the plate. Scoring continued until all worms were deceased. All studies were carried out with the experimenter blind to the conditions (drugs and RNAi constructs) being tested. Furthermore, all results were corroborated by at least 3 separate lifespan curves. Several drugs not reported here significantly increased lifespan in one or more studies but the drugs reported here significantly increased lifespan in at least 3 separate lifespan studies.

In the CL2006 model for proteotoxicity associated with Alzheimer's disease [12], worms were scored daily for paralysis starting at day 1 adult through to day 12–13 at which point all or most of the worms had died from β-amyloid induced toxicity. Since comparison between moribund and paralyzed becomes unreliable as the mortality rate increases, we stopped scoring once mortality exceeded 50%. In the initial screens worms were scored as paralyzed if they failed to respond to prodding in the posterior portion of the body but still moved the head and or pharynx and dead if no movement was observed. Since both paralysis and shortened lifespan are due to A-beta toxicity, subsequent screens simply treated paralyzed and dead animals the same. Nevertheless, scoring was stopped after < 50% survival to maintain consistency. All scoring was performed blind to the drugs being tested.

### RNAi

In these studies either *daf-16* or *cbp-1* was inhibited using RNAi by feeding *C. elegans* bacteria containing dsRNA constructs complementary for the targeted genes [34]. The bacteria expressing these double-stranded RNAi constructs were from Source Bioscience.

### Statistics

Statistical significance of effects on lifespan was determined using the Kaplan-Meier test of survivorship along with the log-rank Mantel-Cox test for median lifespan, as implemented in Prism 4. Statistical significance of effects on age-dependent acceleration of mortality rate as a measure of the rate of aging [17] was determined using a maximum-likelihood method implemented in R, which we have extensively validated [9], [18], [35]. Of particular relevance, we validated this method by demonstrating that it predicts maximal lifespan, 50% survival time, variance in control groups and distribution of deaths better than standard the standard log-linear regression analysis, although the actual values of A and G are very similar when computed by either method [18]. Because of the increased power of this algorithm, it is possible to estimate age-associated acceleration of mortality rate (G) and initial mortality rate (A) using survival curves with substantially fewer individuals than was previously required [18]. One reason the MLE provides substantially more power than log-linear regression is that the latter is unduly influenced by mortality rates at the extremes of lifespan, especially toward maximum lifespan [18]. Similarly, mean lifespan is much more sensitive than median lifespan to the extremes of lifespan, so most standard survival curve statistics, including the log-rank Mantel-Cox, are based on the median rather than mean lifespan. Nevertheless, since many studies report mean lifespan, we include these estimates for the proposed studies as well.

### Supporting information

Multiple drug screens were performed to determine the both the efficacy and optimal concentration of drugs recovered from the FDA approved drug library. Primary screens in *C. elegans* elucidated 6 drugs capable of protecting against amyloid induced toxicity, enhancing lifespan, or both (figure S1). Significant increases in lifespan are observed with caffeine, CPX, tannic acid, acetaminophen, and bacitracin (p  =  or < .05 as measured by Mantel-Cox). Baicalein did not yield significant differences in this experiment. However, treatment with baicalein trends towards an increase in lifespan. Further, the small sample size (n = 15) likely obscures a more significant increase, as these increases in lifespan are observed in several other studies (data not shown).

## Supporting Information

Figure S1 **Survival curves of seven FDA approved drugs discovered to be protective in a** ***C. elegans*** **screen.** Lifespan extension observed with caffeine A. Lifespan extension observed with Ciclopirox olamine (B). Lifespan extension produced by tannic acid (C). Lifespan extension produced by acetaminophen (D). Lifespan extension observed with bacitracin (E). Lifespan extension observed with baicalein (F).
(TIF)
