# Supplementary figures and images for: FDA-Approved Drugs that Protect Mammalian Neurons from Glucose Toxicity Slow Aging Dependent on Cbp and Protect Against Proteotoxicity
Source: PLoS One. 2011 Nov 16;6(11):e27762. doi: 10.1371/journal.pone.0027762 (PMC3218048; doi:10.1371/journal.pone.0027762)

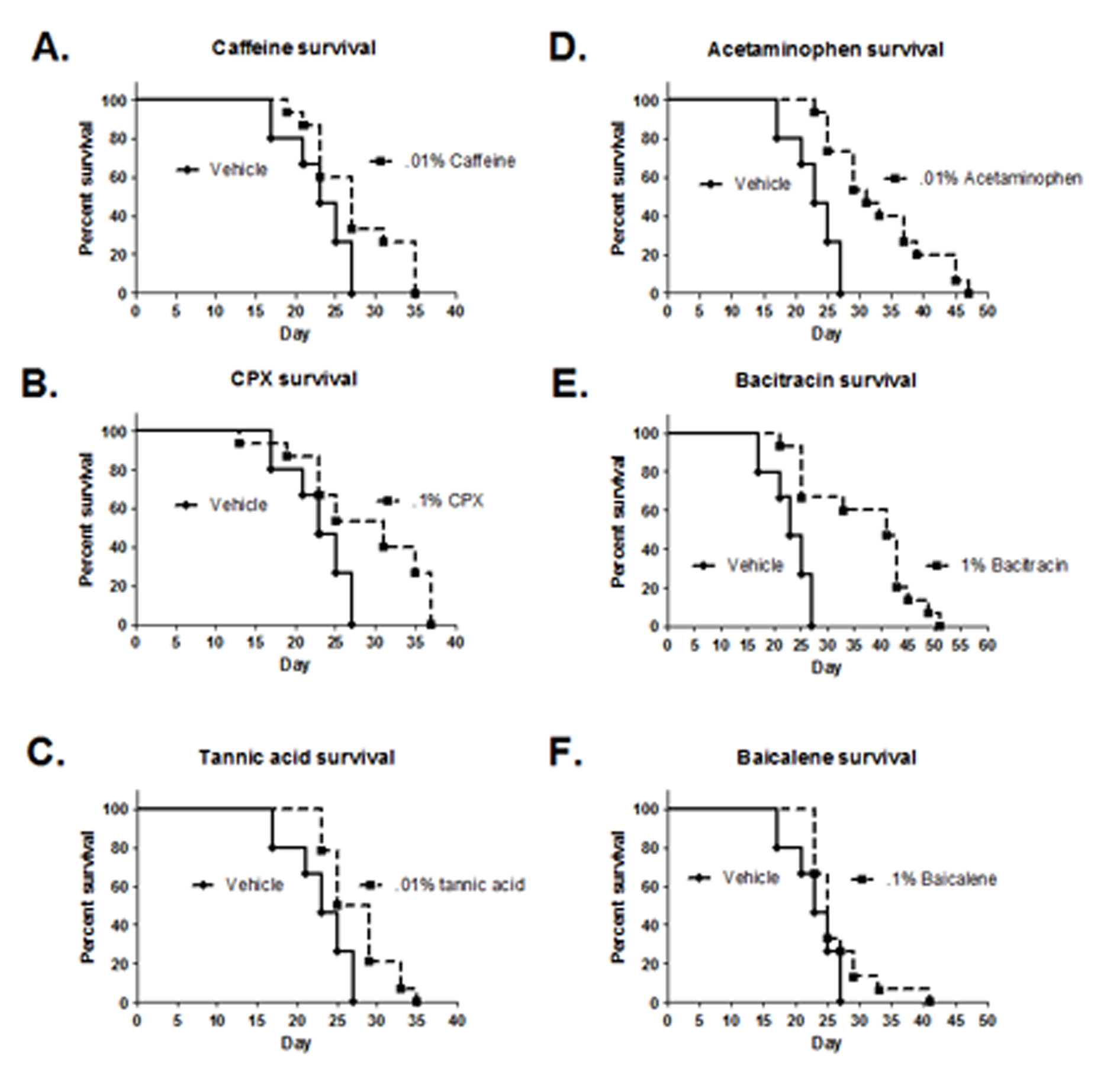

Supplement: Figure S1 — Survival curves of seven FDA approved drugs discovered to be protective in a C. elegans screen. Lifespan extension observed with caffeine A. Lifespan extension observed with Ciclopirox olamine (B). Lifespan extension produced by tannic acid (C). Lifespan extension produced by acetaminophen (D). Lifespan extension observed with bacitracin (E). Lifespan extension observed with baicalein (F). (TIF) [file pone.0027762.s001.tif]
